# Pedogenesis of typical zonal soil drives belowground bacterial communities of arable land in the Northeast China Plain

**DOI:** 10.1038/s41598-023-41401-0

**Published:** 2023-09-04

**Authors:** Meng Hou, Xiaorui Zhao, Yao Wang, Xuemei Lv, Yimin Chen, Xiaoguang Jiao, Yueyu Sui

**Affiliations:** 1 Key Laboratory of Mollisols Agroecology, Northeast Institute of Geography and Agroecology, Chinese Academy of Sciences, 150081 Harbin, People’s Republic of China; 2 University of Chinese Academy of Sciences, 100049 Beijing, People’s Republic of China; 3 College of Modern Agriculture and Eco-Environment, Heilongjiang University, 150080 Harbin, People’s Republic of China

**Keywords:** Biodiversity, Microbial ecology, Soil microbiology

## Abstract

Belowground bacterial communities play essential roles in maintaining ecosystem multifunction, while our understanding of how and why their distribution patterns and community compositions may change with the distinct pedogenetic conditions of different soil types is still limited. Here, we evaluated the roles of soil physiochemical properties and biotic interactions in driving belowground bacterial community composition across three typical zonal soil types, including black calcium soil (QS), typical black soil (HL) and dark brown soil (BQL), with distinct pedogenesis on the Northeast China Plain. Changes in soil bacterial diversity and community composition in these three zonal soil types were strongly correlated with soil pedogenetic features. SOC concentrations in HL were higher than in QS and BQL, but bacterial diversity was low, and the network structure revealed greater stability and connectivity. The composition of the bacterial community correlated significantly with soil pH in QS but with soil texture in BQL. The bacterial co-occurrence network of HL had higher density and clustering coefficients but lower edges, and different keystone species of networks were also detected. This work provides a basic understanding of the driving mechanisms responsible for belowground bacterial biodiversity and distribution patterns over different pedogenetic conditions in agroecosystems.

## Introduction

Soils contribute to basic human needs, such as food, clean air and water, and thus play an important role in sustainable development goals (SDGs)^1^. The soil microbiome regulates vital soil processes, such as aggregation, nutrient cycling and pollutant degradation, all of which are fundamental for soil function, crop productivity and agroecosystem sustainability^2–5^. Different ecological theories have been proposed to explain the potential drivers of belowground biodiversity distribution patterns in topsoil samples at regional or global scales^6–9^. However, as soil is widely thought to be formed by climate conditions, parent material, topography, organisms and time [s = f (c, o, r, p, t)], we speculate that different pedogenetic processes may form soil chronosequences or distinctive soil types that may further shape environmental heterogeneity in soil profiles and determine divergent soil microbial community assembly^10,11^. Soil type forms the habitable ecological niche within the soil and is a combination of soil organic matter, pH, essential nutrients, soil texture and soil aggregations^12^. Changes in soil physiochemical properties can potentially affect soil biodiversity or antibiotic resistant gene profiles among different soil types^13^. However, even though we know that soil type may influence the microbial community structure, patterns of distribution of microbial communities in different soil types, as well as the driving mechanisms responsible for these distribution patterns over different pedogenetic conditions, have not been systematically investigated thus far.

Based on different pedologic conditions (e.g., temperature and precipitation), different soil types are usually presented as zonal distribution patterns along longitudes or latitudes at large spatial scales^14^. Black calcium soil (Chernozems), black soil (Mollisols), and dark brown soil (Alfisols) are three main zonal soil types in the Northeast China Plain. As the different processes of soil formation, including clayization, salinization, desalinization and alkali bleaching, these three zonal types of soil contain distinctive diagnostic characteristics and thus have heterogeneous environmental properties along soil profiles^15,16^. Of these three zonal soil types, Chernozems have a calcic horizon and a higher pH because of the content of CaCO_3_ in the vertical soil profile^14^. Mollisols are one of the typical soil types and are the most fertile and productive soils in China^17^. Alfisols often contain a clay-enriched B horizon in profile with relatively low pH values^18^. Previous studies have provided critical knowledge about the drivers of community assembly features and distribution patterns of soil microbes in the Northeast China region^19,20^. Whereas, much less is known about the mechanisms driving changes in soil biodiversity under distinctive pedogenesis or zonal soil types, though this knowledge is critical for regional soil resource management and sustainable agricultural development in the Northeast China Plain regulated by the soil microbiome.

In terrestrial ecosystems, soil microbial taxa tend to co-occurrence and form clusters that are expected to have ecological implications for community stability and ecosystem multifunctionality^21,22^. Based on the analysis of relationships among the abundances of microbial taxa, we can get more information about the complex interactions between taxa such as mutualism, commensalism, parasitism, predation, and competition^23,24^. Additionally, the topological characteristics of networks, including the node degree, clustering coefficient and modularity can reflect environmental fluctuations and community stability in multiple ecosystems^25,26^. Species in the network with a higher node degree value or centrality can also be statistically identified as keystone taxa^27^. These highly connected taxa can exert a dramatic impact on microbial community ecological network, and their removal may lead to community fragmentation^28^. Recently, studies have demonstrated that the network structure and its topological features could be significantly altered by converse in cropping practices and climate change at local or global scales; thus, these characteristics of ecological networks can be used as indicators of environmental sensitivity^29,30^. However, we could not quantify the relationship between microbial ecological network characteristics and zonal soil types especially in the typical agroecosystem. Improving our understanding of the response of ecological networks features to different soil types may also provide more valuable information about the soil resources management and regional agroecosystem multifunction.

We here hypothesized that belowground bacterial community and its co-occurrence networks might be associated with distinctive soil types that developed under different soil formation processes. To test this hypothesis, we analyzed 9 soil profiles from Chernozems area in Qiqihaer, Mollisols area in Hailun and Alfisols area in Baoquanling across the Northeast China Plain, which is one of the most intensive agroecosystems on the global scale. Topographically, these three soil types were distributed from west to east and also corresponded to the zonality of the increased precipitation. The precipitation gradient was often considered to be one of the most important factors influencing soil formation and the changes in precipitation can alter the abundance and composition of soil microorganisms. The study area in Qiqihaer has lower precipitation than other two sites, and the weak calcium carbonate leaching in the soil is conducive to the formation of chernozems. In Baoquanling area with higher precipitation and stronger clay leaching, occurred lower pH and formed the dark brown soils. Hailun was located in the central region of the Songnen Plain, where the cold temperate continental climate dominates the accumulation of humus in the soil, promoting the formation of black soil. Thus, these zonal soil types are ideal for studying the environmental preferences of bacterial communities that develop from different pedogenesis. We here address three questions: (1) Do bacterial biodiversity and community composition in soil profiles differ in the typical zonal soil types under distinctive pedogenesis? (2) Do co-occurrence network patterns of bacterial communities differ among different soil types? (3) Are bacterial community structures in different soil types driven by similar environmental features?

## Results

### Physiochemical properties of soil samples in typical zonal soil types

**Figure 1:**
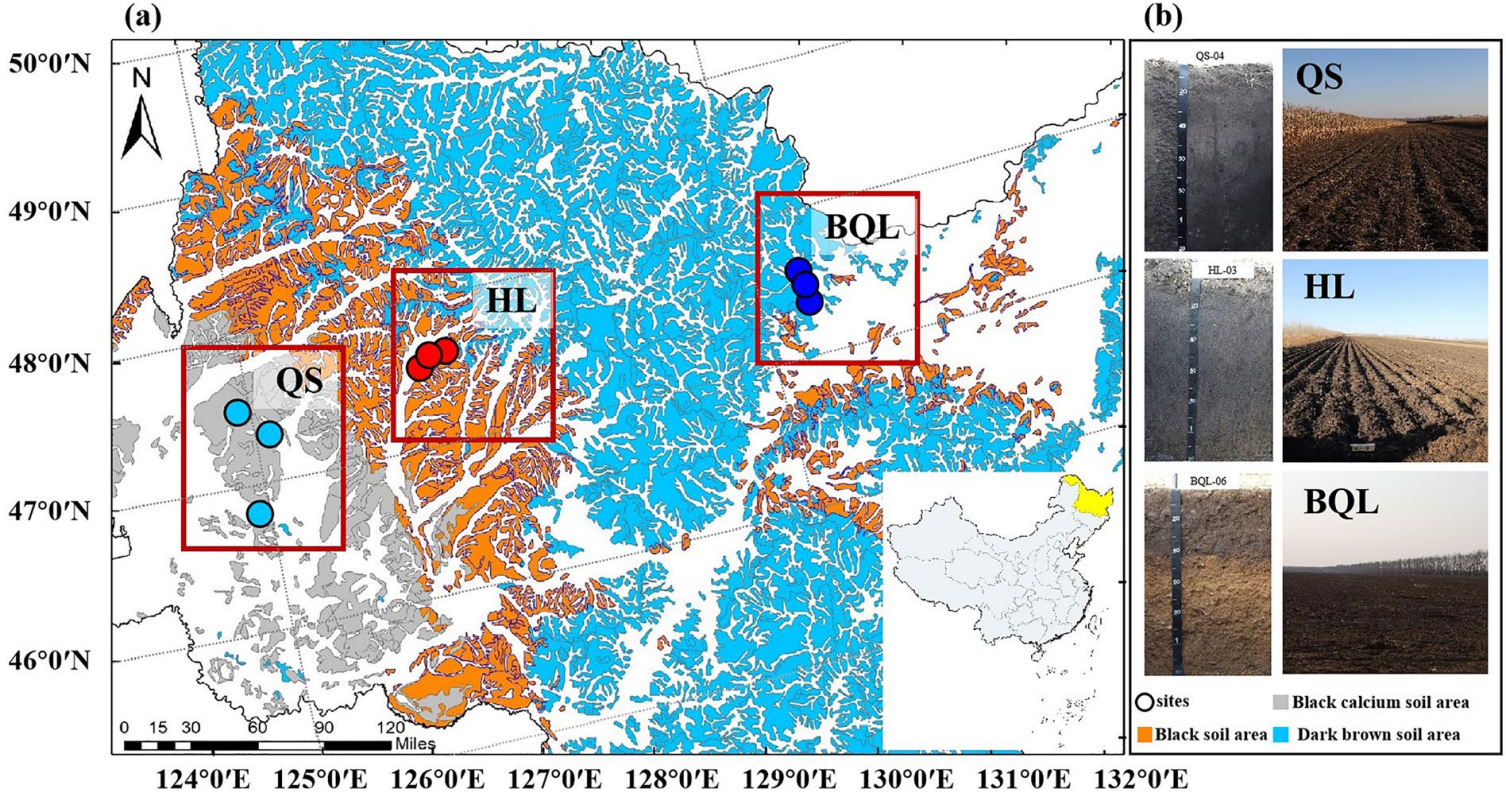
Regional soil map of study area and sampling sites (**a**). Photos of typical soil profiles and landscape (**b**). QS: Black calcium soil sites (in Qiqihaer, China), HL: Black soil sites (in Hailun, China), BQL: Dark brown soil sites (in Baoquanling, China).

Firstly, we summarized the differentiation of soil physiochemical properties under distinct zonal soil type across the Northeast China Plain (Fig. 1 and Table 1). ANOVA revealed that the soil pH of QS sites was significantly higher than that in HL sites and BQL sites (*p* < 0.05), and soil samples from BQL had the lowest pH values (5.95 ± 0.33). For soil physical properties, the results demonstrated that soil samples from BQL sites were more sandy and had higher BD (1.54 ± 0.13 g cm^−3^) (*p* < 0.05). Notably, the content of total K was significantly lower at QS.

**Table 1:** Physiochemical properties of different zonal soil types (mean value ± standard error).

Sample name	Soil type	Statistics	pH	BD (g cm^−3^)	SOC (g kg^−1^)	TN (g kg^−1^)	TP (g kg^−1^)	TK (g kg^−1^)	Sand%	Silt %	Clay %
QS (3 profiles, 13 samples)	Black calcium soil	Mean ± SD	8.13 ± 0.39^a^	1.45 ± 0.09^ab^	9.31 ± 6.05^a^	0.88 ± 0.56^a^	0.40 ± 0.16^a^	31.30 ± 7.64^b^	39.25 ± 9.86^a^	30.35 ± 6.74^b^	30.40 ± 6.00^a^
		Range	7.26–8.61	1.31–1.59	2.74–20.79	0.25–2.02	0.27–0.74	18.65–41.03	25.98–62.39	17.13–37.81	20.49–39.93
HL (3 profiles, 12 samples)	Black soil	Mean ± SD	6.52 ± 0.18^b^	1.37 ± 0.16^b^	16.60 ± 12.24^a^	1.45 ± 0.96^a^	0.53 ± 0.15^a^	46.69 ± 5.55^a^	14.95 ± 10.03^b^	48.47 ± 16.69^a^	36.58 ± 11.03^a^
		Range	6.25–6.81	1.09–1.62	4.40–38.29	0.53–3.24	0.38–0.78	35.83–54.70	3.77–31.92	27.51–81.90	9.40–44.68
BQL (3 profiles, 12 samples)	Dark brown soil	Mean ± SD	5.95 ± 0.33^c^	1.54 ± 0.13^a^	11.02 ± 9.74^a^	1.37 ± 1.31^a^	0.55 ± 0.26^a^	46.73 ± 9.06^a^	51.62 ± 26.23^a^	30.03 ± 18.64^b^	18.36 ± 9.51^b^
		Range	5.42–6.44	1.32–1.71	3.02–26.93	0.45–4.79	0.17–0.99	34.13–64.46	11.46–91.22	1.21–55.93	6.60–32.61

Significant pairwise differences (*p* < 0.05) are denoted with different letters. *BD* soil bulk density, *SOC* soil organic carbon, *TN* soil total nitrogen content, *TP* soil total phosphorus content, *TK* soil total potassium content; Texture cetology: *Sand* soil sand portion, *Silt* soil silt portion, *Clay* soil clay portion.

### Biodiversity and community composition along the typical zonal soil types

**Figure 2:**
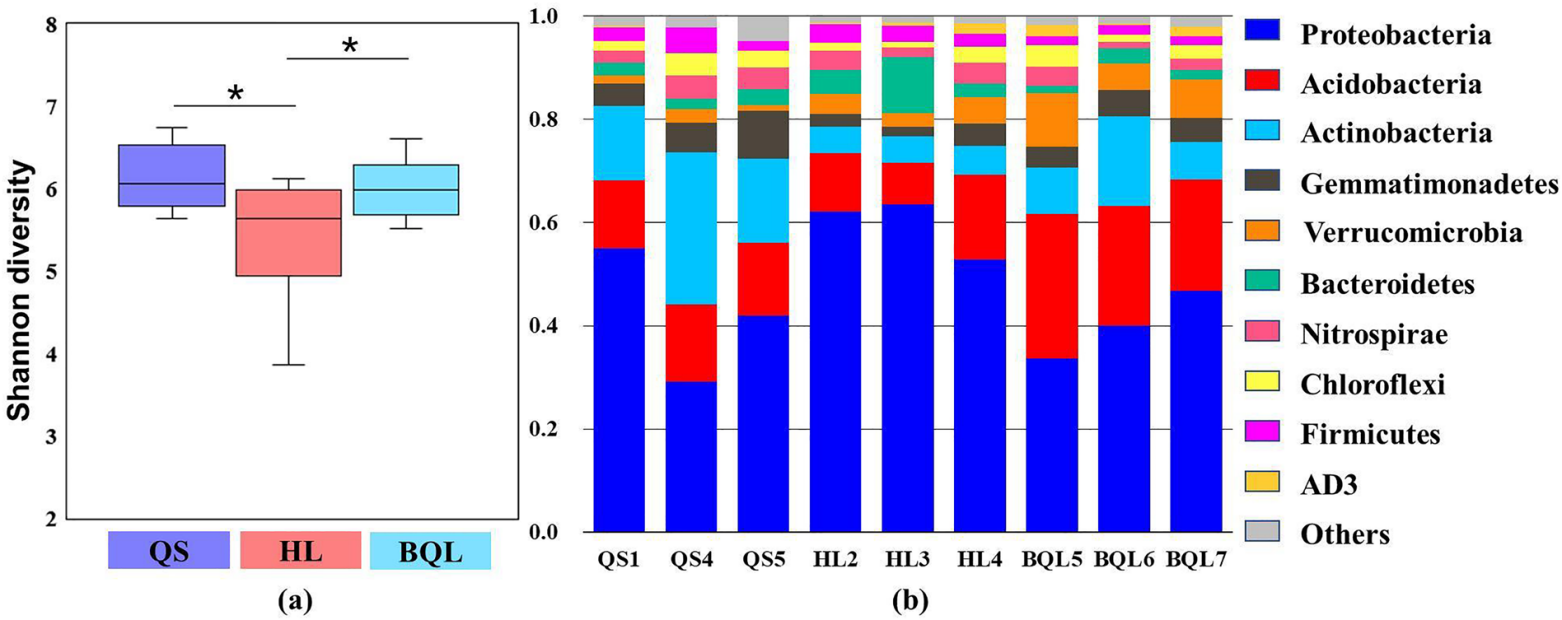
Alpha diversity and relative abundance of soil bacterial communities in HL, QS and BQL. Alpha diversity was calculated using the Shannon index value (**a**). Relative abundance of major taxa (top 10) in the bacterial communities at the phylum level (**b**).

From all 36 soil samples, 2,237,089 high-quality sequences were obtained and clustered into 34,501 operational taxonomic units obtained at 97% sequence similarity. For bacterial alpha diversity, one-way ANOVA indicated that there were significant differences in the bacterial Shannon diversity among different soil types, and sites in HL had significantly lower values than those in BQL and QS (p < 0.05) (Fig. 2a). We also calculated the Simpson index, richness index and Chao 1 of the soil bacterial communities among the different soil types (Table S1).

**Figure 3:**
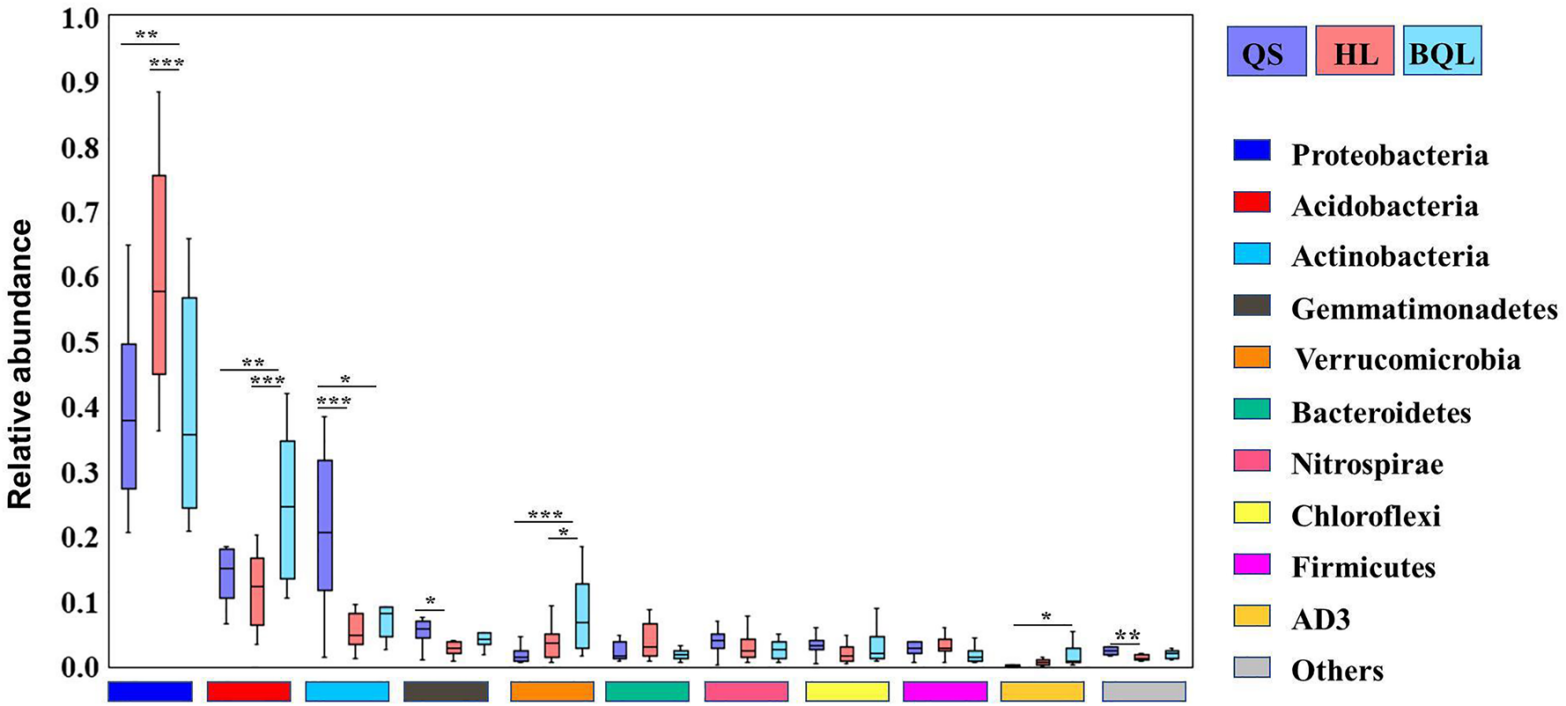
Differences in the relative abundance of the top 10 bacterial taxa at the phylum level among soil types. Significance test: *p < 0.05; **p < 0.01; ***p < 0.001.

These three zonal soil types showed a majority of bacteria from the phyla *Proteobacteria*, *Acidobacteria*, and *Actinobacteria*, and these groups accounted for more than 70% of the bacterial sequences (Fig. 2b). According to the ANOVA analysis, the relative abundance of Proteobacteria was significantly higher in the soil samples of HL sites, which ranged between 39.6 and 59.5% of all sequences. We also found that *Actinobacteria* and *Gemmatimonadetes* were highest in the soil samples from the QS sites and lowest in the soil samples from the HL sites. *Acidobacteria*, *Verrucomicrobia* and *AD3* were significantly enriched in the soil samples from the BQL sites (*p* < 0.05) (Fig. 3).

**Figure 4:**
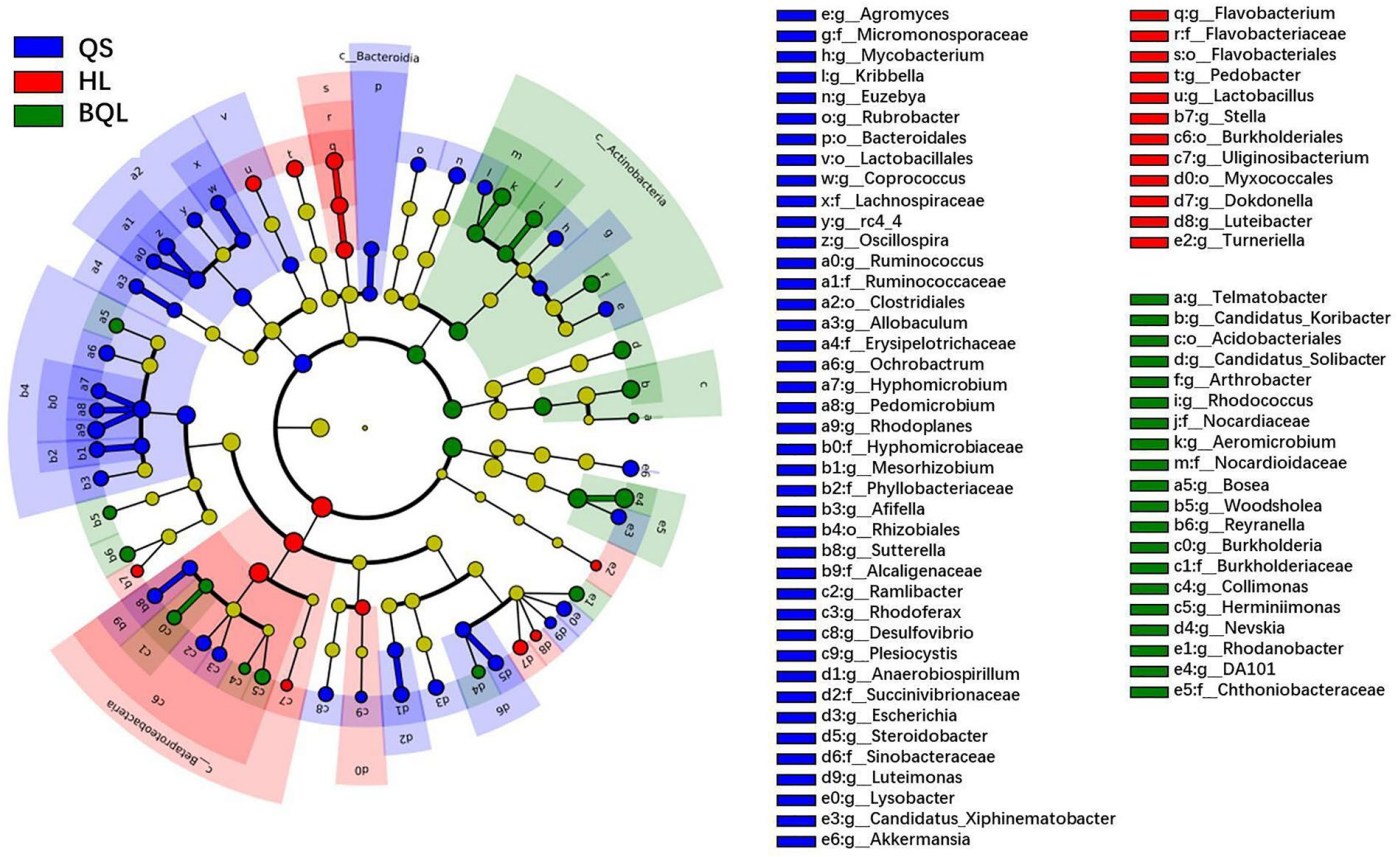
Linear discriminant analysis effect size analysis cladograms of the abundant biomarkers in the typical zonal soil types. Taxa with LDA scores > 3.0 and *p* < 0.05 were identified as biomarkers, and all the detected taxa were assigned to phyla (the outermost), classes, orders, families, genera and species.

Among different soil types, 73 biomarkers with LDA scores greater than 3.0 from different taxonomic levels were detected via linear discriminant analysis effect size (LEfSe) (Fig. 4). The numbers of biomarkers were 41, 12 and 20 in QS, HL and BQL, respectively. In general, the phyla *Proteobacteria*, especially the classes *Alphaproteobacteria* and *Gemmaproteobacteria*, and *Firmicutes* were significantly enriched in QS; meanwhile, *Acidobacteriales* was significantly enriched in BQL. In HL, *Bacteroidetes* (class *Flavobacteriia*, order *Flavobacteriales*, family *Flavobacteriaceae* and genus *Flavobacterium* and class *Sphingobacteriia*, order *Sphingobacteriales*, family *Sphingobacteriaceae* and genus *Pedobacter*), *Firmicutes* (from order to genus *Lactobacillus*), *Proteobacteria* (class *Alphaproteobacteria* (genus *Stella*), class *Betaproteobacteria* (order *Burkholderiales* and genus *Uliginosibacterium*), class *Deltaproteobacteria* (genus *Myxococcales*) and class *Gammaproteobacteria* (genus *Dokdonella* and *Luteibacter*) and *Spirochaetes* (from order to genus *Turneriella*) were dominant compared to other soil types.

**Figure 5:**
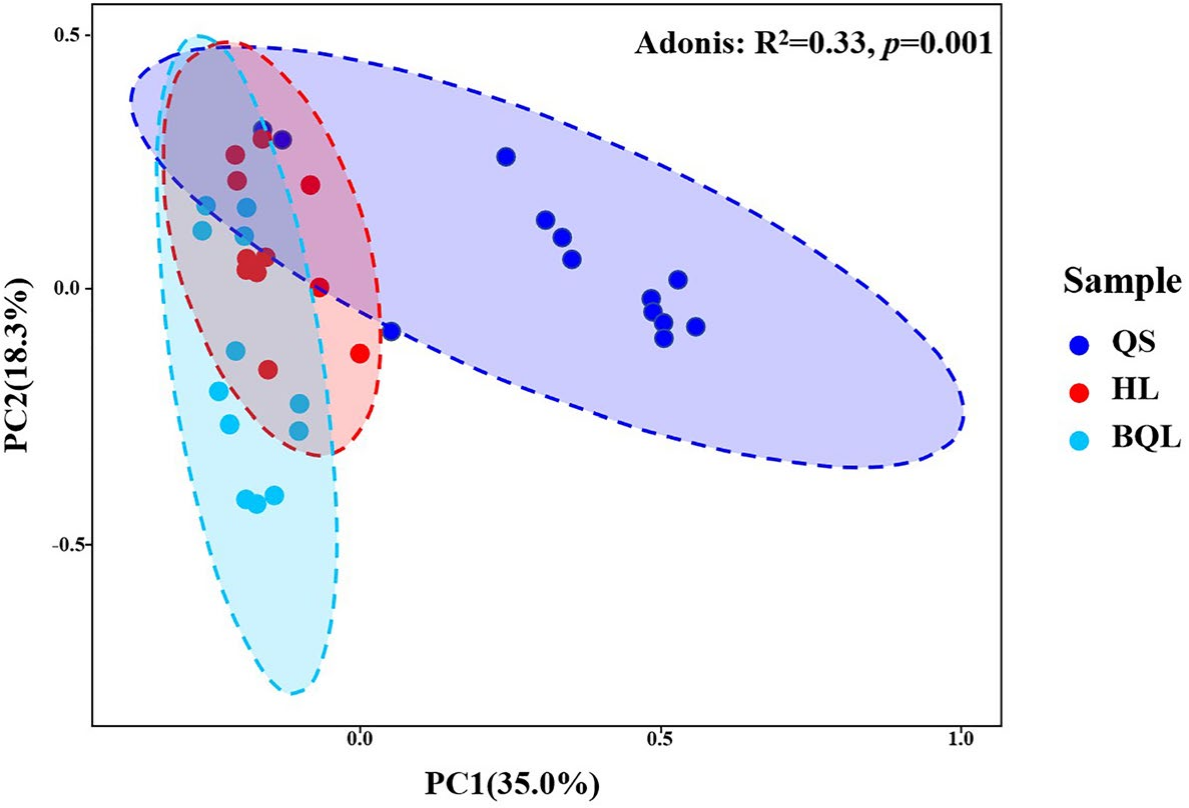
Results of the principal coordinates analysis (PCoA) based on weighted UniFrac distances. The values of axes 1 and 2 are the percentages that can be explained by the corresponding axis. The Adonis analysis result is at the top right corner of the figure. Different colors of the shaded ovals represent samples from different soil types.

An analysis of the Bray–Curtis distance revealed significant differences between the three soil types in terms of bacterial community composition. In the PCoA, PC1 and PC2 explained 35.0% and 18.3%, respectively, of the total variance in bacterial community composition from all soil samples (Fig. 5). The Adonis (*F* = 8.53, R^2^ = 0.33, *p* = 0.001) and PERMANOVA (*F* = 5.96, R^2^ = 0.26, *p* = 0.0049) results both suggested that soil type explained the variation within the bacterial community across these zonal soil types in the Northeast China Plain (All the tested Pairwise dissimilarities of three soil types at the significance level of p < 0.05; Table S2).

### Typical zonal soil co-occurrence networks of bacteria taxa: topological properties and keystone species

**Figure 6:**
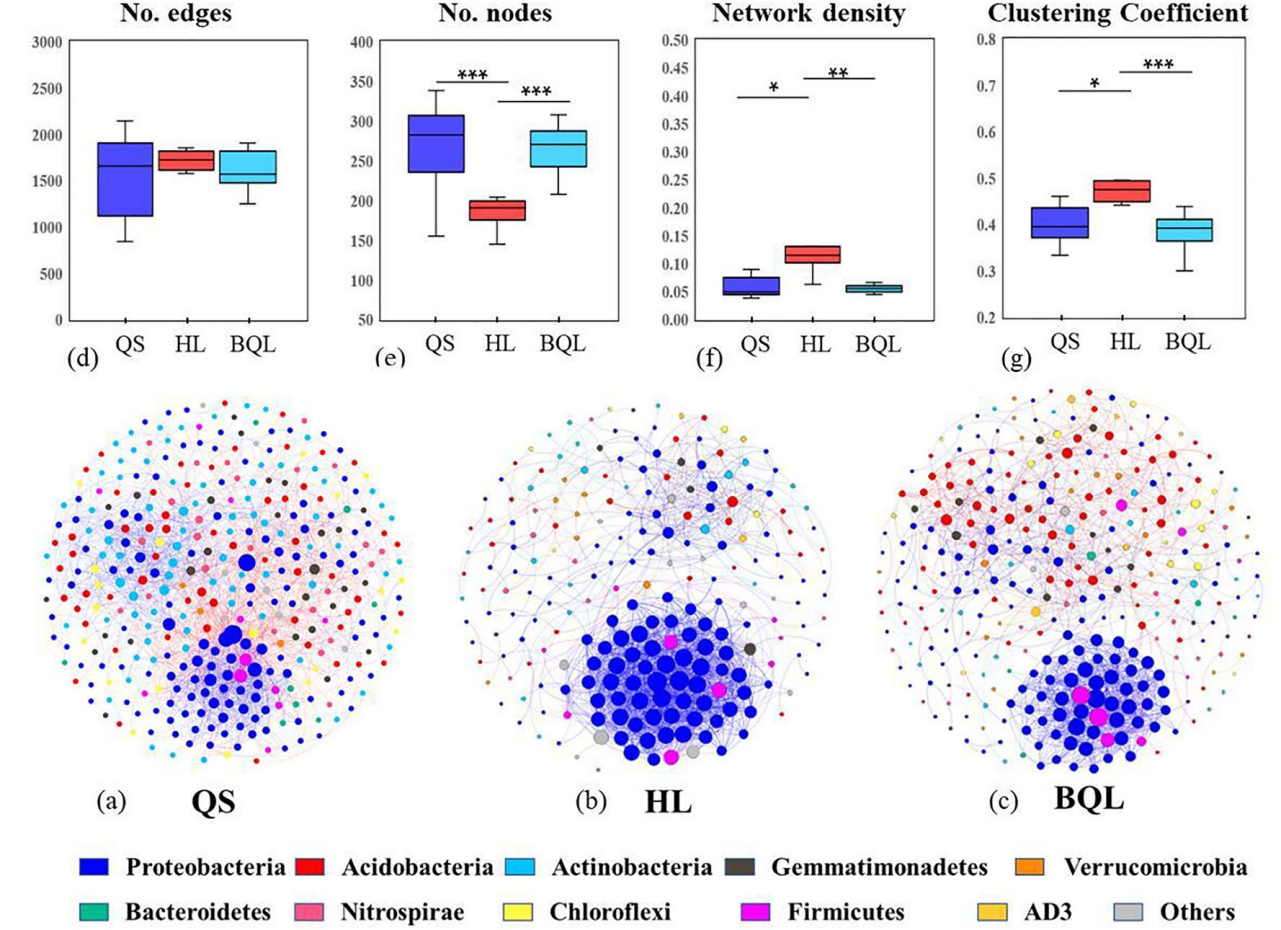
Overview of the co-occurrence networks for bacterial communities in different types of soils (**a**–**c**). Each node represents an Amplicon Sequence Variant (ASV). Node size is proportional to the relative abundance. Edges between nodes represent the relationships among ASVs. Node color represents the different major genera. Blue links indicate positive relationships, and red links indicate negative relationships. Comparison of topological properties in the co-occurrence networks with different soil types (**d**–**f**), and the asterisk indicates that the difference between the two groups is statistically significant.

Using network analysis, we examined the co-occurrence of soil bacterial communities based on strong and significant correlations to clarify the impact of soil type on bacterial species interactions (Fig. 6). It was found that the QS network had more edges and nodes (370 nodes, 2488 edges) than the BQL network (318 edges, 1969 edges) and the HL network (221 edges, 1935 edges) (Fig. 6a–c). We further extracted the subnetwork characteristics of bacterial ecological networks in each soil type to compare the difference in the topological properties of co-occurrence networks in the three types of soil and found that the number of nodes in the HL networks was significantly lower than QS and BQL (Fig. 6d,e). HL network contained the highest ratio of positive to negative correlations, and positive correlations were greater than negative correlations in all three types of soils (Table S3). As a result, the network density and the clustering coefficient of HL were the highest among all networks observed in this study, while BQL was the lowest (Fig. 6f,g), indicating that the network in HL was a more robust microbial co-occurrence network than the other two types of soil.

Furthermore, we identified ASVs with the most connections with other taxa and the highest value of betweenness centrality in the co-occurrence networks, which were potentially identified as keystone taxa in a network. The taxonomy of keystone taxa in the networks was different among soil types (Table 2). These keystone ASVs were primarily derived from *Gammaproteobacteria*, with *Pseudomonas* and *Stenotrophomonas* predominating in BQL and HL, while *Legionellales* and *Bacillus* dominated in QS.

**Table 2:** Network key hubs and taxonomic properties in the QS, HL and BQL (order; family; genus; species).

Sample name	Soil type	OUT ID	Degree	Abundance (%)	Taxonomy (phylum; class; order; family; genus; species)
QS	Black calcium soil	ASV0049	76	0.00366	p__*Proteobacteria*; c__*Gammaproteobacteria*; o__*Legionellales*
		ASV0344	58	0.01533	p__*Proteobacteria*; c__*Gammaproteobacteria*; o__*Enterobacteriales*; f__*Enterobacteriaceae*; g__*Enterobacter*
		ASV0031	54	0.00478	p__*Firmicutes*; c__*Bacilli*; o__*Bacillales*; f__*Bacillaceae*; g__*Bacillus*
HL	Black soil	ASV7234	62	0.16607	p__*Proteobacteria*; c__*Gammaproteobacteria*; o__*Pseudomonadales*; f__*Pseudomonadaceae*; g__*Pseudomonas*; s__*veronii*
		ASV7577	61	0.25279	p__*Proteobacteria*; c__*Gammaproteobacteria*; o__*Xanthomonadales*; f__*Xanthomonadaceae*; g__*Stenotrophomonas*; s__*maltophilia*
		ASV7017	61	0.49401	p__*Proteobacteria*; c__*Gammaproteobacteria*; o__*Enterobacteriales*; f__*Enterobacteriaceae*; g__*Citrobacter*
BQL	Dark brown soil	ASV7070	52	0.11085	p__*Proteobacteria*; c__*Gammaproteobacteria*; o__*Pseudomonadales*; f__*Pseudomonadaceae*; g__*Pseudomonas*; s__*veronii*
		ASV7025	52	0.06301	p__*Proteobacteria*; c__*Gammaproteobacteria*; o__*Pseudomonadales*; f__*Moraxellaceae*; g__*Acinetobacter*; s__*johnsonii*
		ASV7361	52	0.15905	p__*Proteobacteria*; c__*Gammaproteobacteria*; o__*Xanthomonadales*; f__*Xanthomonadaceae*; g__*Stenotrophomonas*; s__*maltophilia*

### Pedogenesis of distinct soil types driving the assembly of bacterial community and special members

**Figure 7:**
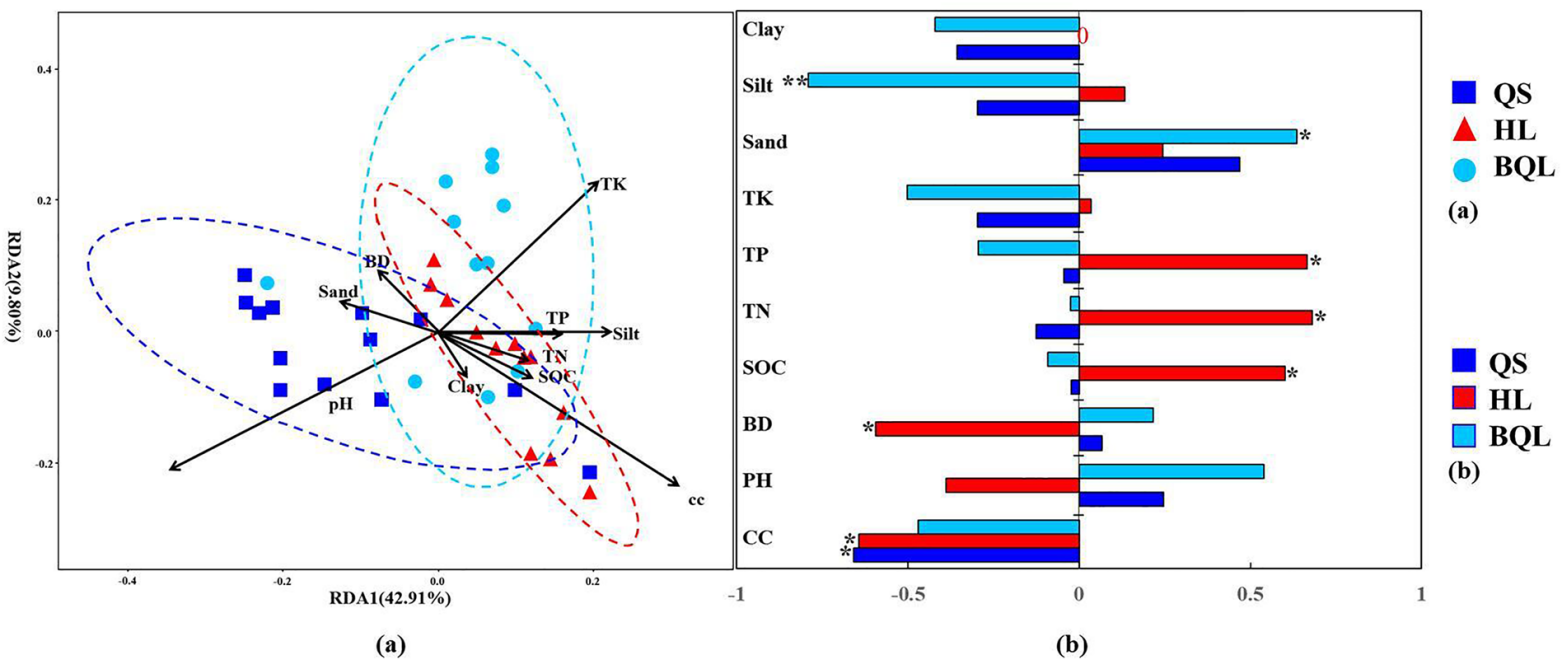
Redundancy analysis (RDA) indicating the relationships between environmental variables and soil bacterial community structure among different zonal soil types (**a**). Spearman’s correlation coefficients between bacterial community composition (axis NMDS1) and soil properties for individual soil types (**b**). *BD* soil bulk density, *SOC* soil organic carbon, *TN* soil total nitrogen content, *TP* soil total phosphorus content, *TK* soil total potassium content, *CC* network clustering coefficient, *Sand* soil sand portion, *Silt* soil silt portion, *Clay* soil clay portion. Significance test: *p < 0.05.

According to the RDA result, soil physicochemical properties strongly affected the composition of bacterial communities under different soil types (Fig. 7a). Among soil types, the driving factors of soil bacterial communities differed. For QS samples, among the soil properties, bacterial communities were strongly correlated with pH and soil sand portion. However, the contents of SOC and TN in HL were the main driving factors for bacterial community composition. Concentration of total K in BQL soil samples was significantly correlated with bacterial communities composition. And the contributions of soil properties to variations in bacterial community structures indicated that soil pH, TK, TP, soil silt portion and soil bacterial network clustering coefficient had significant effects on the bacterial community structures (R^2^ = 16.56–46.83%, *p* < 0.05) (Table S4). Spearman correlations between bacterial community composition (value of NMDS1) and soil properties in three types of soil showed that soil nutrients, including the contents of SOC, TN and TP, were most significantly positively correlated with the bacterial community composition in HL samples (Fig. 7b) (*p* < 0.05).

**Figure 8:**
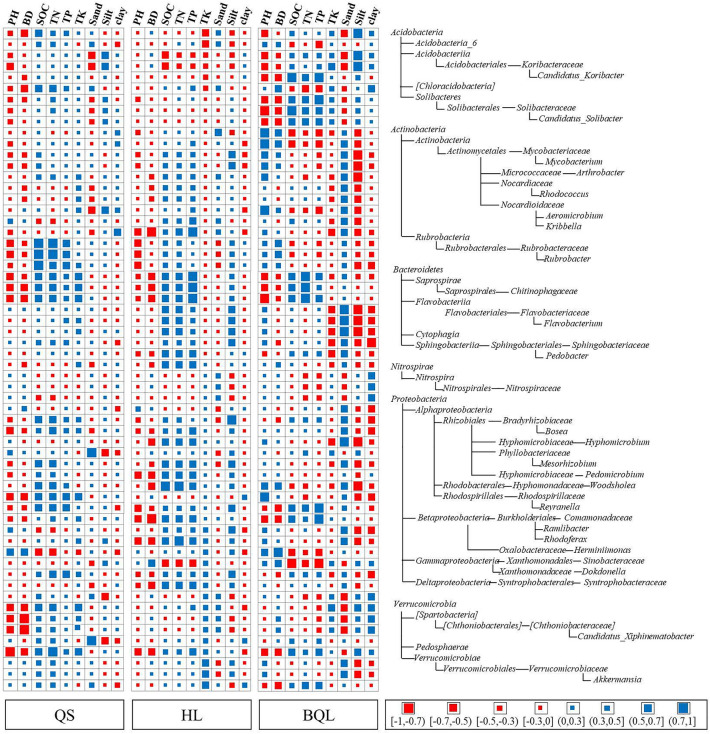
Heatmap of Spearman’s rank correlation coefficients between relative abundances of the enriched bacterial taxa from phyla to genera and soil properties in three typical types of soil. The square size and color represent the magnitude and direction of the correlation coefficient, respectively, as indicated in the figure legend. The same taxa and their sublevels were merged into one line/row on the heatmap.

We further calculated bacterial correlation coefficients between taxa relative abundance and soil properties. According to Spearman's rank correlation coefficients (ρ), strong (|ρ| > 0.7), moderate (0.7 ≤ |ρ| < 0.5), weak (0.5 ≤ |ρ| < 0.3), and negligible (|ρ| ≤ 0.3) associations were identified^31,32^. All 60 bacterial taxa selected from the phylum to genus level had strong or moderate correlations with more than one soil physiochemical property, which were different among the three major soil types (Fig. 8). And these taxa had higher relative abundance and differed significantly across the three types of soil. In total, there were strong or moderate correlations between bacterial taxa and soil properties in the BQL samples, especially in *Acidobacteria* and its sublevel taxa. There was a strong positive correlation between *Saprospirales*, *Rubrobacterales*, and *Alphaproteobacteria* species and soil nutrients, including SOC, TN, TP, and TK, but a negative correlation between bulk density and soil pH for the QS and HL samples. For samples in BQL, soil texture, including silt and clay portions, was strongly negatively correlated with the taxa *Actinobacteria*, *Flavobacteriia*, *Alphaproteobacteria* and *Verrucomicrobiae*. The details of the Spearman correlation rank coefficient and *p* value between the relative abundance of soil bacterial taxa and soil characteristics in different types of soil was shown in the Table S5.

## Discussion

Considering the effects of soil types on belowground biodiversity and community composition, previous studies have often been limited to examining topsoil or focused on only one type of soil^33^. In spite of this, it may be difficult to explore the effect of soil types on bacterial communities only in the topsoil due to the complex interaction between the parent materials, historical pedological conditions, and current anthropogenic activities. The vertical soil profile recorded the legacy of soil-forming processes, including diagnosed features and soil properties represented by horizon notation, suggesting both past and present change of soil processes^34^. Thus, we here systematically compared the diversity, community composition and co-occurrence network patterns of bacteria along the vertical soil profiles in the typical zonal soil types of black calcium soil (Chernozems), black soil (Mollisols), and dark brown soil (Alfisols). Bacterial communities in different soil types exhibited distinctive and complex environmental preferences and vulnerabilities.

### Distinctive pedogenesis of typical zonal soil types determine the physiochemical properties

The composition of soil microbial communities is influenced by a number of edaphic factors, such as pH, nutrient levels, organic C storage, moisture availability, and soil texture^35^. These edaphic factors typically vary with soil type, so we should expect strong corresponding shifts in microbial community structure under different soil types^12^. In this study, we selected three typical soil types, Chernozems, Mollisols, and Alfisols, to perform profile excavation and sample collection; these soil types are sequentially distributed from west to east on the Northeast China Plain. The results demonstrated that nearly all the soil physicochemical properties were, to some extent, changed with soil types, but only features of pH, bulk density and soil texture showed significant differences (Table 1), which may have been caused by the changes in soil properties such as the content of SOC, TN and TP in different soil layers of vertical soil profiles, hiding their significance of differences among soil types.

Pedogenetic features, such as illuviation, eluviation and redox reactions, determine the amount, reactivity, and vertical distribution of available nutrient absorption within soil types^36^. In this study, soil pH variability in chernozems is generally limited to the range of 7.26–8.61, which is higher than those of black soil (6.25–6.81) and dark brown soil (5.42–6.44) (Table 1). Chernozems were formed under the transition from temperate subhumid to semiarid and meadow steppe plants with obvious humification and calcification^37^. Chernozems are distributed in the western part of our study area (Fig. 1) with lower precipitation and higher temperatures; thus, the accumulation of soil humus is weak, and the thickness of the humus layer is thinner than that of the adjacent black soil^38^. Humification is the most important pedogenetic process during Mollisol formation. Under the temperate subhumid climate, coupled with the long cold winter, the decomposition of organic matter by microorganisms was limited in black soil^39^. Therefore, the accumulation of humus content is strong, as embodied in the deep humus layer and relatively high concentration. Mollisols contained higher levels of SOC, TN, and TP than the other two types of soil. The soil-forming process of Alfisols is mainly characterized by the accumulation of weakly acidic humus matter, mild leaching and clayization^18^. Before the dark brown soil area was transformed into farmland, the natural vegetation was mixed broadleaf-conifer forest, with lush herbaceous vegetation under the forest and lower pH values. This region experiences simultaneous heat and precipitation, which may result in a large amount of litter remaining on the surface each year as the bioaccumulation process occurs.

### Soil types have a great effect on the bacterial biodiversity

Due to the high heterogeneity of soil physiochemical properties based on distinctive pedogenesis, soil type is considered the major driver influencing soil bacterial community structure^40^. The soil physicochemical properties of different soil types can significantly influence bacterial and fungal community diversity and structure^41,42^. In our study, the Shannon index of HL was significantly lower than that of QS and BQL, while the organic carbon and nitrogen contents of HL were the largest among these three types of soils, suggesting that the alpha diversity did not increase with higher organic matter contents in these soils. In contrast, analysis of bacterial beta diversity showed clear differentiation among QS, HL and BQL. The HL bacterial communities fall between two clusters of microbiomes belonging to QS and BQL, which corresponded to the geographical locations of the zonal soil types.

We also found that the bacterial communities in these three types of soil were dominated by three major groups (*Proteobacteria*, *Acidobacteria* and *Actinobacteria*) (> 5% of all sequences), which corresponded with the 135 soil samples observed in the North China Plain^43^. Additionally, bacteria afflicted in *Bacteroidetes* were also enriched in HL. It has been reported that *Bacteroidetes* are usually copiotrophs and are most abundant in soils with high levels of labile organic carbon storage^44^. The relative abundance of *Proteobacteria* may decrease significantly when nutrient deficiencies are present as some species of Proteobacteria prefer nutrient enrich habitats^45^. Typical black soil showed relatively high C, N, and P contents during the humification process, which may explain why the abundance of *Proteobacteria* was significantly higher in HL than in QS and in BQL. *Verrucomicrobia* and *Bacteroidetes* are two groups that participate in soil C cycling, and both follow a parabolic pattern with increasing N rates, as do *Nitrospirae*^46^. In this study, their relative abundances in HL and BQL were higher than those in QS. There are many *Acidobacteria* species found in soil samples throughout the terrestrial ecosystems, including *Acidobacteriaceae*, which is capable of adapting to varied environments and using a variety of polysaccharides^47^. These *Acidobacteria* are usually acidophilic and less likely to compete in the more neutral soil pH environment and thus can be enriched in the acidic soil samples of BQL. *Actinobacteria* and *Gemmatimonadetes* were detected in the black soils of China, as they can drive SOM degradation processes^48,49^. In this study, *Actinobacteria* and *Gemmatimonadetes* were enriched in QS, which may be attributed to the fact that the black calcium soil region had lower precipitation and a higher rate of soil organic matter turnover. However, the relative average of *Chloroflexi* in the grass courses was nearly 10% (North Carolina, USA)^50^, which is five times higher than the abundances of the phyla in this study. Some *Chloroflexi* genera (e.g., *Anaerolinea*, *Caldilinea*, *Roseiflexus*) are obligate anaerobes capable of degrading cellulose^51^, which may explain why *Chloroflexi* were seldom found in this study's soils.

### Network analysis revealed the effects of soil types on bacterial interactions

Using microbial co-occurrence network to predict how changes in the environment may affect agroecosystem functions is a promising approach^52^. Through network analysis, previous studies have determined that soil type is the major factor determining the composition and interaction of microorganisms across multiple ecosystems^53,54^. We here found that significant difference of the clustering coefficient and network density of co-occurrence networks between HL and QS and BQL, which indicated that HL had a more stable bacterial community network. In these three types of soils, more edges and nodes were grouped in the bacterial network of QS than in the bacterial networks in BQL. It has been suggested that the neutral pH range is the optimal environment for most microbes^55^. Because of the lower pH values, network of BQL soil samples had loosed network structure with a lower clustering coefficient in these constructed co-occurrence networks, whereas Zhang et al.^43^ reported that bacterial phylotype richness and phylogenetic diversity decreased with the pH decreasing (pH 4–7).

There is evidence that soil texture plays a significant role in shaping the community of soil microbes^31^. The bacterial diversity in clay and silt fractions was also found to be higher than in sand fractions due to higher nutrient availability in fine particles and protection from predation in small pores^56^. However, HL had higher silt and clay fractions than the other types of soil, but had significantly lower bacterial α-diversity, potentially indicating that the soil texture had different influences on bacterial diversity in the case of high soil nutrient content. Positive correlations may indicate cooperative or mutualistic potential associations such as cross-feeding and/or syntrophic relationships, while negative correlations could infer antagonistic associations among species such as predation and/or competition for a limiting resource^57,58^. There were the highest ratios of positive correlations to negative correlations in HL, which suggesting that the black soils increased bacterial cooperation. In soil samples with high pH values, we assumed that the finer texture would provide sufficient substrate to promote microbial growth, ultimately forming a co-occurrence network with a less competitive structure for the microbial community.

Through network construction, we can gain new insights into microbial ecological interactions and the responses of microbial network structure to environmental changes in ecosystem^59^. Highly connected taxa in the network, which usually consider as keystones are essential for maintaining the functions of soil microbial ecosystems^60^. In our research, the keystone taxa in the network of the three soil types were mostly members of *Proteobacteria*, which is the phylum enriched in these soils (Fig. 6 and Table 2). The keystone taxa in the bacterial networks of HL and BQL were affiliated with *Gammaproteobacteria*, while QS also contains members of *Firmicutes*, partly because of their high abundance. Plant performance has been reported to be improved by *Gammaproteobacteria* enriched, which can fix nitrogen and produce hormones. They are often considered to be important groups of microbes that colonize the rhizosphere^61^. Given the similar network keystone taxa of bacterial communities in HL and BQL, these two types of soil might have some similar chemical properties (Table 1). *Pseudomonas* and *Stenotrophomonas* were found to be the main keystone nodes in BQL and HL, and it has been reported that these microbial genera play an important role in plant growth and nutrient cycling, especially in the rhizosphere and root compartments^62^. It has been demonstrated that *Stenotrophomonas* can produce indoleacetic acid, which stimulates plant growth and photosynthesis^63^, and many *Pseudomonas* species play a beneficial role in plant phenotypic plasticity^64^. A noteworthy *Bacillus* taxon was observed in QS, and Egamberdiyeva^65^ showed that *Bacillus* can efficiently stimulate NPK uptake in maize plants and would grow better in soils that were deficient in nutrients over soils that were abundant in nutrients. Different soil types enriched numerous genera, such as *Bacillus*, *Sphingomonas*, and *Pseudomonas*. There was a high proportion of these taxa in soil communities and they were important for plant growth and soil restoration^66^. The role of keystone taxa in microbial communities will be affected by soil physicochemical properties along distinctive soil formation processes, and thus different types of soil may further affect bacterial diversity^67,68^.

### Different environmental parameters related to pedogenesis shaped bacterial communities in the three types of soils

Understating the factors controlling microbial diversity in typical soil types in the Northeast China Plain is critical to the development of sustainable food production and environmental sustainability. Among different soil types, soil physiochemical properties may create specific niches and exert environmental selection forces to shape the composition of microbial communities. To find more ecological implications between the bacteria and their preferences to inhabit the three types of soils, we systematically compared soil characteristics, including pH, BD, SOC, TN, TP, TK, and soil texture, under different soil types. The driving forces of the bacterial community in these three soil types were also different. We speculated that this differentiation was caused by the distinctive soil formation processes.

For explaining the variance in bacterial community structures, soil pH is the most representative characteristic of various parent materials of soil chemical parameters, as reported in numerous studies, both on an experimental scale and on a global scale^69^. In this study, pH was the main factor influencing soil bacterial communities in QS. The calcification in QS may potentially explain the relationship between pH and the bacterial community. For Mollisols, the dark humus-rich surface horizons formed by humification generally contain higher nutrient levels. This supported that bacterial communities in Mollisols were significantly correlated with soil nutrient contents, including SOC, TN and TP. After soil pH, soil texture has been considered to be the second most important factor shaping soil microbial communities, as well as a predictor of soil hydrophobicity^50^. Furthermore, soil texture (clay or silt content) was found to be a significant influence on phylum-level bacterial distribution in French soils^70^. In this study, particle size distribution was analyzed to explore the association between bacterial communities and soil texture across typical soil types in the northeast plain. Here, we found that soil texture may directly impose a physiological constraint on the bacterial community in BQL. This may also reflect the long-lasting process of eluviation and illuviation in BQL, which may lead to cation losses that are coarser along the profiles.

Environmental parameters affect community structure differently at a whole-community level depending on soil properties, suggesting that parameter effects vary greatly based on taxa and geographical scale. At the phylum level, soil texture (clay or silt content) was considered the major driver of bacterial distribution^70^. As a further investigation of the relationship between those taxa and environmental factors, we conducted correlation analysis and found that some bacterial taxa showed strong correlations with environmental factors in BQL but weak correlations in QS and HL. A possible explanation is that BQL soil was acidic and had a lower pH, as well as a higher sand content than clay because of the process of clayzation. It may also have been due to the fact that higher agronomic inputs to increase crop yields provided sufficient substrate for the bacterial community's growth, thus weakening the correlation between their abundances and soil physicochemical properties as compared to natural soils^71^.

As mentioned above, distinct types of soil can enrich divergent bacterial species. A Spearman correlation analysis found a negative correlation between *Acidobacteria* abundance and soil pH (Fig. 8). According to previous studies, *Acidobacteria* thrive in relatively acidic soils and are sensitive to pH changes^72,73^. *Actinobacteria* and their sublevel taxa were primarily regulated by texture^74^, and our results showed that the relative abundances of *Arthrobacter* and *Mycobacteriaceae were* strongly negatively correlated with the soil silt portion in BQL. Although different correlations patterns have been found in QS and HL, these differences may be explained by differentiation in soil types and heterogeneity physiological characteristics^75^. It has been shown that members of *Proteobacteria* and *Bacteroidetes* are copiotrophic^44,76^ and function as initial metabolizers of soil organic matter which can be enriched in soils that contain high levels of nitrogen and carbon^77,78^ The results of our study consistently showed positive correlations between *Bacteroidetes* (e.g., *saprospirae*) in HL and the content of SOC, TN, and TP. Additionally, SOC, TN, and TP were also correlated with taxa from *Rhizobiale* and *Burkholderiales*.

## Conclusion

As a summary, this study compared three typical types of zonal soils in the Northeast China Plain and belowground bacterial communities response to different soil pedogenetic conditions. Our findings suggest that different soil types had distinct microbial biomasses and community compositions and structures. Higher soil nutrient contents were accompanied by biodiversity and greater bacterial co-occurrence connectivity in typical black soil, opposite to the results obtained from the black calcium soil and dark brown soil. In typical black soil, microbial co-occurrence patterns were less complex, but more stable than in black calcium soil and dark brown soil. Different keystone species were identified within soils among the soil types. There were three key factors influencing the bacterial community composition and structure in black calcium soil, typical black soil, and dark brown soil, respectively: pH, SOC, and soil texture. These findings demonstrate the importance of considering the soil type when developing sustainable agriculture and maintaining microbially driven agroecosystem diversity in the study area.

## Materials and methods

### Sample collection

Soil profiles were investigated in arable land with three typical soil types under a rotation of maize and soybeans in Heilongjiang province, China in the 2020s (Fig. 1). The three typical soil types were selected from west to east, which were classified as Black calcium soil (QS), Typical black soil (HL) and Dark brown soil (BQL) according to the Genetic Soil Classification of China (GSCC), as classified as Cal-ustic Isohumisols, Hapli-Udic Isohumosols, Mol-boric argosols in the Chinese Soil Taxonomy (CST) respectively, and as Chernozems, Mollisols and Alfisols in the US Soil Taxonomy (ST)^15,79^. Profiles description including location, crops in season and landscape photograph are available in Appendix A in Supplementary materials. The mean annual temperature of QS, HL and BQL is 4–5 °C, 0.5–4 °C and − 2 to 5 °C respectively, and the rainfall of approximately is about 350–500 mm, 500–600 mm and 500–700 mm. A total of 37 samples from 9 soil profiles of cultivated land were selected based on soil type and its distribution, average temperature and precipitation (Fig. 1). Soil samples were subsequently taken to the laboratory with packages of ice within 12 h, then sieved over 2 mm. All samples were divided into two sections, one stored at – 80 °C for biological analysis and the other at 4 ℃ for physicochemical property analysis.

### Soil physicochemical properties analyses

Soil texture were categorized in sand portion (200–2000 μm), clay portion (50–200 μm) and silt portion (2–50 μm) by using the sedimentary analysis. Soil pH was measured by a pH meter (Mettler Toledo FE20, Shanghai, China) after stirring the soil water (1:2.5; w/v) in suspension for 30 min. Soil bulk density (BD) was estimated based on the weight and volume of the soil cores (100 g cm^−3^) after subtracting those of the rocks and roots of the plants. Oxidation of potassium dichromate with soil organic carbon (SOC) was used to measure SOC (HJ615–2011, Chinese Standards for Determination of Soil Organic Carbon). With an Elemental analyzer (VarioEL III, Germany) soil total nitrogen content (TN) was determined. With the molybdenum-blue method, the total P (TP) content of soil was measured after digestion with hydrofluoric acid-perchloric acid. By using a flame photometry instrument (FP640, Shanghai Precision Instrument Co., Shanghai, China), the soil potassium content was analyzed with hydrofluoric perchloric acid.

### High-throughput sequencing

Total DNA was extracted from 0.5 g soil using the FastDNA SPIN Kit for soil (MP Biomedicals, Solon, OH, USA) as manufacturer protocol. With a NanoDrop 2000 spectrophotometer (Thermo Fisher Scientific, Waltham, MA), DNA concentration and its purity were measured. We conducted PCR amplification of the bacterial 16S rRNA genes V3–V4 region using primer 341F (5′-CCTAYGGGRBGCASCAG-3′) and reverse primer 806R (5′-GGACTACNNGGGTATCTAAT-3′). In all PCR reactions, 15 µL of Phusion® High-Fidelity PCR Master Mix (New England Biolabs) were used; A total of 2 µM forward and reverse primers were used, as well as approximately 10 ng of template DNA (Wekemo Tech Group Co., Ltd. Shenzhen China). In this thermal cycling procedure, the denaturation temperature was set at 98 °C for 1 min, then cycling it through 30 cycles of denaturation at 98 °C for 10 s, annealing at 50 °C for 30 s, elongation at 72 °C for 30 s, and a final 72 °C for 5 min. Apply electrophoresis on 2% agarose gel after mixing the 1XTAE buffer with the PCR products in the same volume. A mixture of PCR products at equal densities was prepared. Next, mixture PCR products were purified using Qiagen Gel Extraction Kit (Qiagen, Germany). According to manufacturer's recommendations, sequencing libraries were generated using TruSeq® DNA PCR-Free Sample Preparation Kit (Illumina, USA). Index codes were added after the sequencing libraries were generated. A Qubit@2.0 Fluorometer (Thermo Scientific) was used to assess the quality of the library. Lastly, Illumina NovaSeq was used to generate 250 bp paired-end reads from the library.

### Bioinformatics and statistical analyses

Raw data of FASTQ files are converted into a format that can be operated by the QIIME2 pipeline (https://docs.qiime2.org/2019.1/). A feature table of Amplicon Sequence Variants (ASVs) was constructed from demultiplexed sequences from each sample by quality filtering, trimming, denoising, merging and identifying chimeric sequences^80^. The database of GREENGENES (13_8) was used for taxonomic classification^81^.

### Network analyses

Using CoNet plugin in Cytoscape (3.7.1), we constructed 3 co-occurrence networks between bacterial taxa across all soil samples in three soil types to better understand bacterial connectivity^52^. We filtered the ASVs in detail by setting > 2/3 of the samples in each network as the minimum occurrence, and then evaluated pairwise associations using the methods of Pearson, Spearman, Bray–Curtis, and Kullback–Leibler. The topological parameters of each constructed co-occurrence network were analyzed using Network Analyzer that plugin within Cytoscape. Network graph was visualized on the platform of Gephi (0.9.2)^82^ for network visualization.

### Statistical analyses

All statistical analyses for soil characteristics and bacterial diversity were performed using R platform (v 3.6.2)^83^ and SPSS (v17.0) (IBM Corp., Chicago, IL, USA). Using Spearman's rank-order correlation, we examined the relationships between bacterial relative abundance and soil properties, and presented by heatmap using R. For the multiple comparisons of soil properties among soil types and treatments, one-way ANOVA was used along with the post-hoc Tukey's test. Principal Coordinate Analysis (PCoA) was conducted on the Bray–Curtis dissimilarity for bacterial communities from different soil types using the R package ‘ade4’. The significance of compositional differences among the three soil types was further assessed using the Adonis and permutational multivariate analysis of variance (PERMANOVA) method with pseudo-F statistic. This was conducted using the R package ‘vegan’.

Using a linear discriminant analysis Effect Size (LEfSe) method, biomarkers with different abundances were identified on the Galaxy platform (https://huttenhower.sph.harvard.edu/galaxy/)^84,85^. For each sample, Shannon diversity indices were used to characterize alpha diversity. Principal coordinate analysis (PCoA) was used to visualize the structural variation in microbial communities across samples using beta-diversity distance measurements, including Bray–Curtis distance, unweighted UniFrac, and weighted UniFrac^86^. Using the R package 'vegan', we performed redundancy analysis (RDA) to analyze microbial communities' relationships with environmental factors^87^.

### Supplementary Information

Supplementary Information 1.

Supplementary Table S1.

Supplementary Table S2.

Supplementary Table S3.

Supplementary Table S4.

Supplementary Table S5.

## Data Availability

The sequence data were uploaded to Sequence Read Archive (SRA) of National Center for Biotechnology Information (NCBI) as accession number SRP374605 (https://www.ncbi.nlm.nih.gov/sra/?term=SRP374605).
