# Supplementary material for: Pedogenesis of typical zonal soil drives belowground bacterial communities of arable land in the Northeast China Plain
Source: Sci Rep. 2023 Sep 4;13:14555. doi: 10.1038/s41598-023-41401-0 (PMC10477331; doi:10.1038/s41598-023-41401-0)
Supplement: Supplementary file 1 — Supplementary Information 1. [file 41598_2023_41401_MOESM1_ESM.docx]

**Appendix A**

**The detail of soil profiles sites and photos of soil profiles and landscape**

| **Soil type** | **Sample name** | **Location** | **Latitude and longitude** | **Altitude (m)** | **crop** |
| --- | --- | --- | --- | --- | --- |
| Black calcium soil | QS-1 | Lindian, Daqing | 47.318930°N，125.127094°E | 161 | Soybean |
|  | QS-4 | Fyyu, Qiqihaer | 47.519443°N，124.868113°E | 158 | Soybean |
|  | QS-5  HL | Fyyu, Qiqihaer | 46.803390°N，124.887088°E | 152 | Soybean |
| Black soil | HL-2 | Haibei, Hailun | 47.639923°N，126.798029°E | 210 | Soybean |
|  | HL-3 | Aimin, Hailun | 47.553561°N，126.686425°E | 198 | Soybean |
|  | HL-4 | Zhayinhe, Hailun | 47.629715°N，126.967287°E | 249 | Soybean |
| Dark brown soil | BQL-5 | Shangzhi, Baoquanling | 47.487907°N，130.492316°E | 105 | Maize |
|  | BQL-6 | Shangzhi, Baoquanling | 47.520450°N，130.481979°E | 115 | Maize |
|  | BQL-7 | Duluhe, Baoquanling | 47.560028°N，130.473593°E | 116 | Maize |


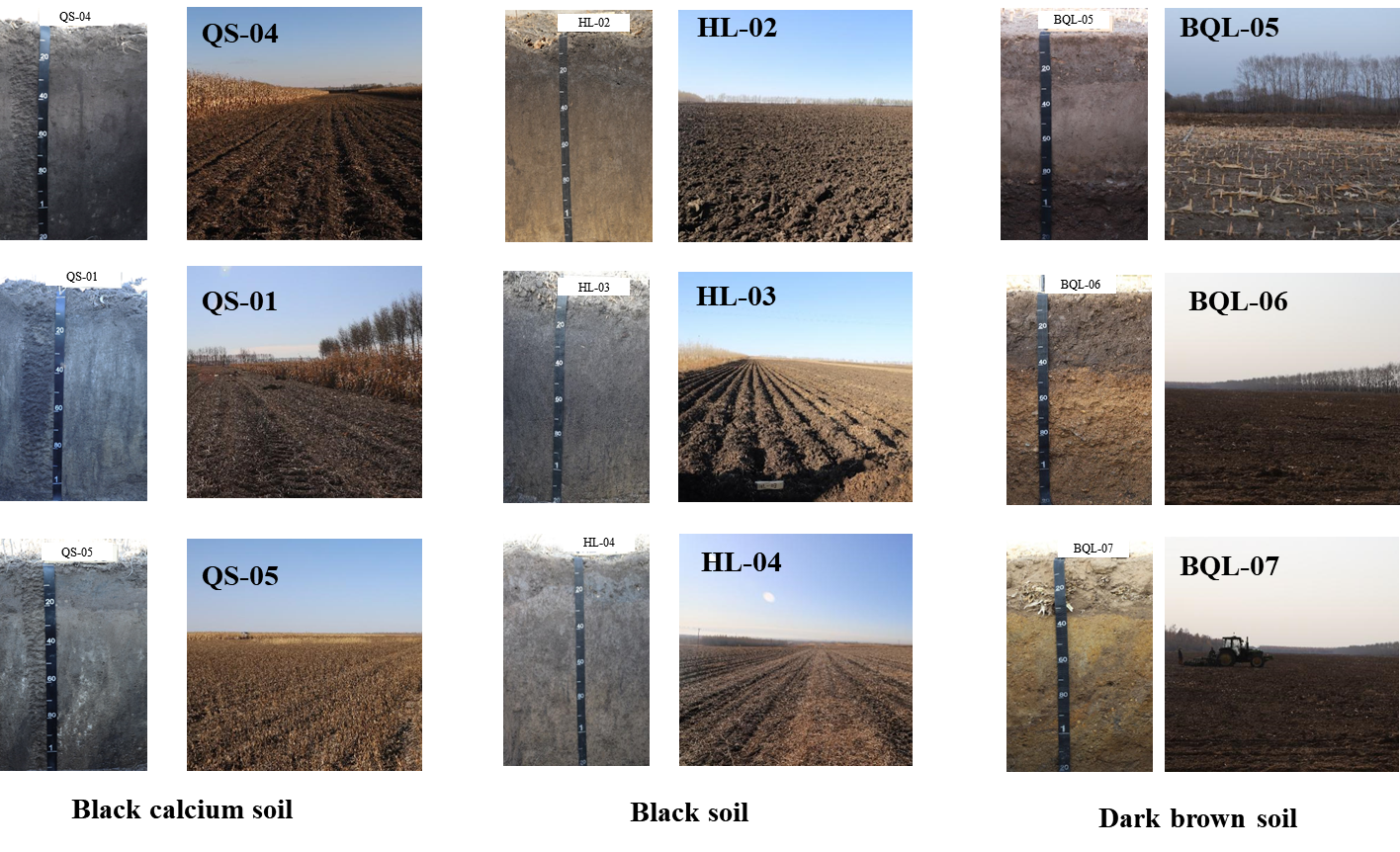


Soil profiles excavated with the scale of 1.2 m wide, 2-4 m long, and 1–2 m deep, and three replicates soil profiles were collected from three soil types region randomly. Then soil profiles were divided into different soil layers according to diagnostic characters. and the soil samples were collected from the middle parts of each soil profile layers away from the plant root system.

**The full description of nine soil profiles was shown in the table below:**

| Genetic Horizon | Depth (cm) | Munsell Color | | | | Texture* | Structure (%) | | | Bulk Density |
| --- | --- | --- | --- | --- | --- | --- | --- | --- | --- | --- |
|  |  | Dry | | | Moist |  | Sand  2-0.05 | Silt  0.05-0.002 | Clay  0.05-0.002 | g/cm^3^ |
| **Profile QS-1. Black calcium soil** | | | | | | | | | | |
| Apk | 0-24 | 4/1 10YR | 3/1 10YR | | | Clay loam | 26.0 | 37.0 | 37.0 | 1.41 |
| Ahk | 24-35 | 5/1 10YR | 4/2 10YR | | | Clay loam | 31.6 | 37.8 | 30.6 | 1.37 |
| Bk | 35-86 | 7/1 10YR | 4/2 10YR | | | Clay loam | 29.3 | 36.7 | 34.0 | 1.31 |
| Ck | 86-125 | 7/2 10YR | 5/2 10YR | | | Clay loam | 38.1 | 34.6 | 27.3 | 1.52 |
| **Profile QS-04. Black calcium soil** | | | | | | | | | | |
| Ap | 0-20 | 4/2 10YR | 2/3 10YR | | | Loam | 42.6 | 35.0 | 22.4 | 1.36 |
| Ah | 20-41 | 5/2 10YR | 3/2 10YR | | | Clay loam | 39.7 | 26.1 | 34.2 | 1.37 |
| Bk1 | 41-65 | 6/2 10YR | 3/3 10YR | | | Clay loam | 37.1 | 23.0 | 39.9 | 1.37 |
| Bk2 | 65-102 | 6/2 10YR | 4/2 10YR | | | Clay loam | 31.9 | 37.1 | 31.0 | 1.57 |
| Bk3 | 102-127 | 5/2 10YR | 4/2 10YR | | | Clay loam | 34.9 | 27.5 | 37.6 | 1.59 |
| **Profile QS-05. Black calcium soil** | | | | | | | | | | |
| Ap | 0-22 | 5/2 2.5YR | 3/2 10YR | | | Sandy clay loam | 62.4 | 17.1 | 20.5 | 1.49 |
| ABk | 22-45 | 5/2 10YR | 3/3 10YR | | | Sandy clay loam | 51.1 | 23.9 | 25.0 | 1.52 |
| Bk | 45-80 | 7/1 10YR | 5/2 10YR | | | Sandy clay loam | 47.5 | 26.1 | 26.4 | 1.42 |
| Bkt | 80-120 | 6/2 10YR | 4/3 10YR | | | Clay loam | 38.1 | 32.6 | 29.3 | 1.55 |
|  |  |  |  | | |  |  |  |  |  |
| **Profile HL-02. Black soil** | | | | | | | | | | |
| Ap | 0-21 | 3/2 10YR | 2/1 10YR | | | Clay | 20.8 | 37.7 | 41.5 | 1.09 |
| Ah | 21-55 | 4/2 10YR | 2/2 10YR | | | Clay | 29.0 | 28.4 | 42.6 | 1.31 |
| AB | 55-90 | 5/2 10YR | 2/3 10YR | | | Silty clay | 7.9 | 47.4 | 44.7 | 1.46 |
| BC | 90-118 | 5/2 10YR | 3/3 10YR | | | Silty clay | 3.8 | 53.8 | 42.4 | 1.48 |
| **Profile HL-03. Black soil** | | | | | | | | | | |
| Ap | 0-25 | 3/1 10YR | 2/1 10YR | | | Silty loam | 11.4 | 70.9 | 17.7 | 1.20 |
| Ah | 25-67 | 4/1 10YR | 3/1 10YR | | | Clay | 24.1 | 33.9 | 42.0 | 1.28 |
| AB | 67-93 | 4/2 10YR | 2/1 10YR | | | Silty clay loam | 7.0 | 54.8 | 38.2 | 1.53 |
| BC | 93-125 | 6/3 10YR | 3/4 10YR | | | Silty clay loam | 5.1 | 56.2 | 38.7 | 1.62 |
| **Profile HL-04. Black soil** | | | | | | | | | | |
| Ap | 0-23 | 4/2 10YR | 2/2 10YR | | | Silty loam | 8.7 | 81.9 | 9.4 | 1.18 |
| Ah | 23-52 | 5/2 10YR | 3/2 10YR | | | Clay | 31.9 | 27.5 | 40.6 | 1.34 |
| AB | 52-70 | 6/2 10YR | 2/3 10YR | | | Silty clay | 7.2 | 52.2 | 40.6 | 1.43 |
| BC | 70-103 | 5/3 10YR | 3/4 10YR | | | Clay | 22.4 | 37.0 | 40.6 | 1.51 |
| C | 103-125 | 6/3 10YR | 4/3 10YR | | | ---- |  |  |  | 1.57 |
| **Profile BQL-05. Dark brown soil** | | | | | | | | | | |
| Ap | 0-26 | 6/3 10YR | | 2/3 10YR | | Clay loam | 28.7 | 41.1 | 30.2 | 1.62 |
| Br | 26-55 | 7/3 10YR | | 5/3 10/YR | | Clay loam | 24.8 | 47.8 | 27.4 | 1.58 |
| Btr | 55-73 | 6/3 10YR | | 4/4 10/YR | | Silty clay loam | 11.5 | 55.9 | 32.6 | 1.57 |
| BCrg | 73-87 | 7/3 10YR | | 3/4 10YR | | Sand clay loam | 64.6 | 15.7 | 19.7 | 1.57 |
| C | 87-120 | 6/4 10YR | | 5/4 10YR | | Loamy sandy | 88.5 | 0.19 | 0.96 | 1.55 |
| **Profile BQL-06. Dark brown soil** | | | | | | | | | | |
| Ap | 0-24 | 4/4 10YR | 2/3 10YR | | | Loamy sandy | 84.1 | 9.3 | 6.6 | 1.32 |
| Ah | 24-37 | 3/4 10YR | 2/3 10YR | | | Sandy loam | 55.7 | 37.4 | 6.9 | 1.51 |
| C | 37-122 | 7/6 10YR | 6/6 10YR | | | Sandy | 97.7 | 0.8 | 1.6 | 1.71 |
| **Profile BQL-07. Dark brown soil** | | | | | | | | | | |
| Ap | 0-32 | 5/4 10YR | 3/4 10YR | | | Loam | 40.0 | 39.1 | 20.9 | 1.36 |
| Bgr | 32-79 | 7/4 10YR | 5/8 10YR | | | Clay loam | 39.0 | 33.4 | 27.6 | 1.64 |
| Br | 79-99 | 6/4 10YR | 4/4 10YR | | | Loam | 36.2 | 47.6 | 16.2 | 1.66 |
| Cr | 99-120 | 6/4 10YR | 5/6 10YR | | | Sandy loam | 55.2 | 29.7 | 15.1 | 1.68 |
